# Increasing selection gain and accuracy of harvest prediction models in Jatropha through genome-wide selection

**DOI:** 10.1038/s41598-021-93022-0

**Published:** 2021-06-30

**Authors:** Adriano dos Santos, Erina Vitório Rodrigues, Bruno Galvêas Laviola, Larissa Pereira Ribeiro Teodoro, Paulo Eduardo Teodoro, Leonardo Lopes Bhering

**Affiliations:** 1 A&E Statistical Analysis and Consulting, Brasília, DF Brazil; 2 Life and Earth Sciences, Universidade de Brasília - Campus Planaltina, Brasília, Distrito Federal Brazil; 3 Genetics and Biotechnology Laboratory, Embrapa Agroenergia, Brasília, Distrito Federal Brazil; 4 Department of Agronomy, Universidade Federal Do Mato Grosso Do Sul, Chapadão Do Sul, Mato Grosso Do Sul Brazil; 5 Department of General Biology, Universidade Federal de Viçosa, Viçosa, Minas Gerais Brazil

**Keywords:** Agricultural genetics, Plant breeding, Plant genetics

## Abstract

Genome-wide selection (GWS) has been becoming an essential tool in the genetic breeding of long-life species, as it increases the gain per time unit. This study had a hypothesis that GWS is a tool that can decrease the breeding cycle in Jatropha. Our objective was to compare GWS with phenotypic selection in terms of accuracy and efficiency over three harvests. Models were developed throughout the harvests to evaluate their applicability in predicting genetic values in later harvests. For this purpose, 386 individuals of the breeding population obtained from crossings between 42 parents were evaluated. The population was evaluated in random block design, with six replicates over three harvests. The genetic effects of markers were predicted in the population using 811 SNP's markers with *call rate* = 95% and minor allele frequency (MAF) > 4%. GWS enables gains of 108 to 346% over the phenotypic selection, with a 50% reduction in the selection cycle. This technique has potential for the Jatropha breeding since it allows the accurate obtaining of GEBV and higher efficiency compared to the phenotypic selection by reducing the time necessary to complete the selection cycle. In order to apply GWS in the first harvests, a large number of individuals in the breeding population are needed. In the case of few individuals in the population, it is recommended to perform a larger number of harvests.

## Introduction

In recent decades, there has been exponential growth in demand for energy sources, which is linked to population expansion. It is estimated that the world population in 2050 will be 9.7 billion people, compared to approximately 7.3 billion people in 2015, i.e., the population will increase by about 32%^1^. This scenario has imposed challenges on society, as pertinent questions arise: How to increase the production of food to meet society's demand and still meet the environmental sustainability goals? In this context, science is the main ally, since it is possible to develop innovation and technologies to improve yields and restore natural balances throughout the food system simultaneously^2^.

Energy from fossil fuels is still crucial in the energy sector, but it is also known to be the primary source of greenhouse gas emissions and a finite source^3^. Renewable energy has grown fast in recent years, driven by policy support, advances in technology, and sharp reductions in production costs, and is at the heart of the transition to a less carbon-intensive and more sustainable energy system. In this context, using renewable energy is imperative in the world energy matrix. It is worth mentioning the use of biofuels, which has shown economic viability and some advantages compared to fossil fuels since they are non-toxic, biodegradable, and do not pollute the environment, have flash point and can be added to diesel due to similar properties^4^.

In Brazil, there are several potential sources of oilseeds for biodiesel production. Given the vast diversity of the national ecosystem, soybean (*Glycine max* L. Merril) presents highlight and supremacy as feedstock for biodiesel production, representing 69.8% of the Brazilian energy matrix^5^. Thus, there is a limitation in the number of raw materials composing the energy matrix. However, Brazil has the potential to expand the production of biofuels and other vegetable oil derivatives to meet both the domestic and global markets. One of the effective ways to increase the limited quantity of traditional raw materials and their high prices is to invest in the improvement of biodiesel production from inedible vegetable oil^6^, such as Jatropha (*Jatropha curcas* L.).

*Jatropha* has been the target of several studies as a potential source for biodiesel production^7,8^, since it has high oil production (30–48%)^9,10^, rusticity^11,12^ and simple spread^10^. Due to these characteristics, Jatropha qualifies as a potential candidate for the sustainable production of biofuels^13^. Despite the enormous potential, this species is in a domestication stage in Brazil, and because it is perennial, it needs several years to complete a breeding cycle. One of the objectives of the perennial plant breeders is to shorten the selection cycle. For this purpose, an alternative that has been used with considerable success is Genome-Wide Selection (GWS), which is one of the promising tools to increase selection efficiency, reduce costs in the launching of cultivars, reduce the breeding cycle through early selection, and increase the genetic gain between breeding generations^14,15^. For crops like Jatropha, it is estimated that GWS could shorten the breeding cycle^14^, which would have a high impact on the release of new cultivars for planting.

However, few studies have reported the use of GWS in Jatropha worldwide. A pilot evaluation of predictive model accuracy using only one harvest and demonstrated the potential of GWS in Jatropha breeding^16^. However, these authors recommended that the study should be validated over the years and by progeny evaluation. This research had a hypothesis that GWS is a tool that can decrease the breeding cycle in Jatropha. Thus, the objectives of this study were (1) to use the Jatropha training population evaluated in multiple harvests, and (2) to develop models for predicting breeding values between harvests. This paper presents unprecedented results in validating GWS in Jatropha for biofuel production.

## Results

Initially, we estimated heritability in the restricted sense (h^2^_a_) for grain yield in the three harvests, to assess the extent to which phenotypic variation is genetically controlled and genomic selectable. Heritabilities ranged from 0.18 to 0.20 for the first and third harvests, respectively, and 0.25, when the average grain yield was considered (Table 1).

**Table 1:** Narrow-sense heritability (h^2^_a_), phenotypic selection accuracy (r_yy_), predictive ability (r_yg_), and average grain yield (µ).

Harvests	h^2^_a_	r_yy_	µ (g plant^−1^)	r_yg_
I	0.18	0.17	173.76	0.22
II	0.19	0.20	760.85	0.24
III	0.20	0.37	1075.52	0.54
Average grain yield	0.25	0.37	667.09	0.57

Low selective accuracy was observed based on phenotypic information (r_yy_) on all harvests. Even using the mean of harvests, high magnitude accuracy values were not obtained^17^. The values varied from 0.17 to 0.37 for the first and third harvests, respectively.

On the other hand, the GWS (Table 2) accuracies were from low to high magnitude, ranging from 0.20 to 0.83 for the first and third harvests, respectively. As for the GWS analysis efficiency regarding phenotypic selection, when we apply genomic selection in the second year, gains of 66.74% become possible. Likewise, when genomic selection is performed in the third year, gains of 346% in relation to phenotypic selection are achieved.

**Table 2:** Accuracy and efficiency of genomic selection compared to selection only with phenotypic data in Jatropha.

Harvests	GWS accuracy	Efficiency	IRPS (%)
1	0.20	–	–
2	0.31	1.667	66.74
3	0.83	4.464	346.45
Average grain yield	0.80	4.303	330.31

IRPS: Increase relative to phenotypic selection.

Regarding the estimate of the number of individuals required to obtain the desired selective accuracy (Table 3), it is observed the need to evaluate a larger number of individuals when larger estimates of selective accuracy are sought. In an antagonistic way, a smaller number of individuals will be necessary when the number of harvests to obtain accuracy is increased. Regarding the estimate of 0.8, which is considered as high magnitude^17^, it will be necessary to evaluate 1239 and 256 in the first and third harvests, respectively.

**Table 3:** Number of individuals required to obtain the desired accuracy of GWS in the Jatropha population for grain yield.

Desired accuracy	Number of individuals required			
	Harvest 1	Harvest 2	Harvest 3	Average grain yield
0.40	207	175	43	51
0.50	310	263	64	77
0.60	465	394	96	115
0.70	723	613	149	179
0.80	1239	1051	256	307
0.90	2788	2364	576	692

In this case, it can be observed that to obtain high accuracy in GWS requires a large number of individuals. However, this fact is only justified if the trait under study has low heritability, however, if the trait has high heritability, the number of individuals can be reduced. This is evident when we look at Eq. (5), in which a direct relationship between the desired accuracy of GWS and heritability can be seen.

**Figure 1:**
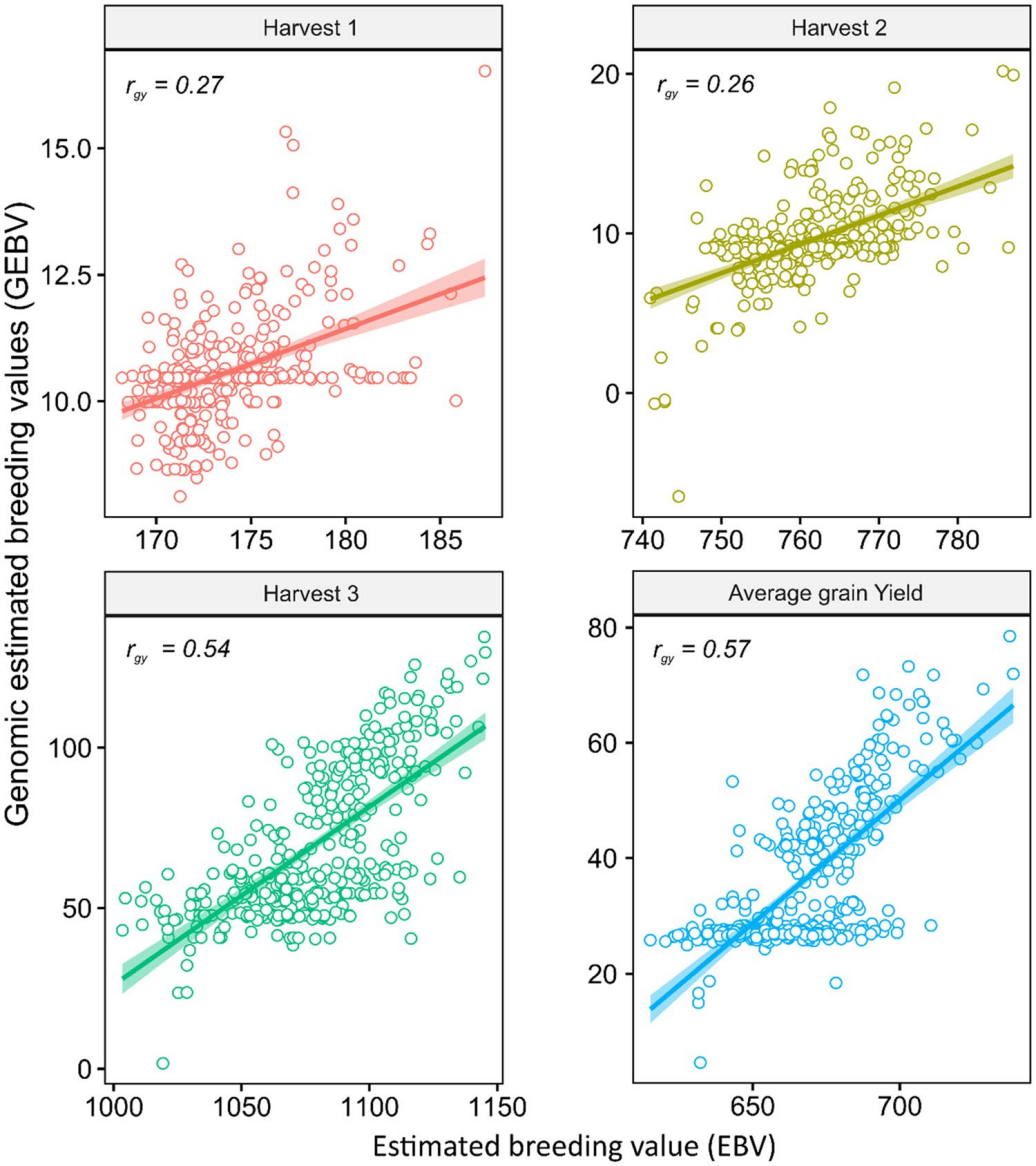
Scatterplots of genomic estimated breeding values (GEBVs) by RR-BLUP and unregressed phenotypes observed for grain yield. r_gy_: predictive ability of genomic selection. The package used of R to create this Figure was ggplot2 (v0.3.3, https://cran.r-project.org/web/packages/ggplot2/index.html).

**Figure 2:**
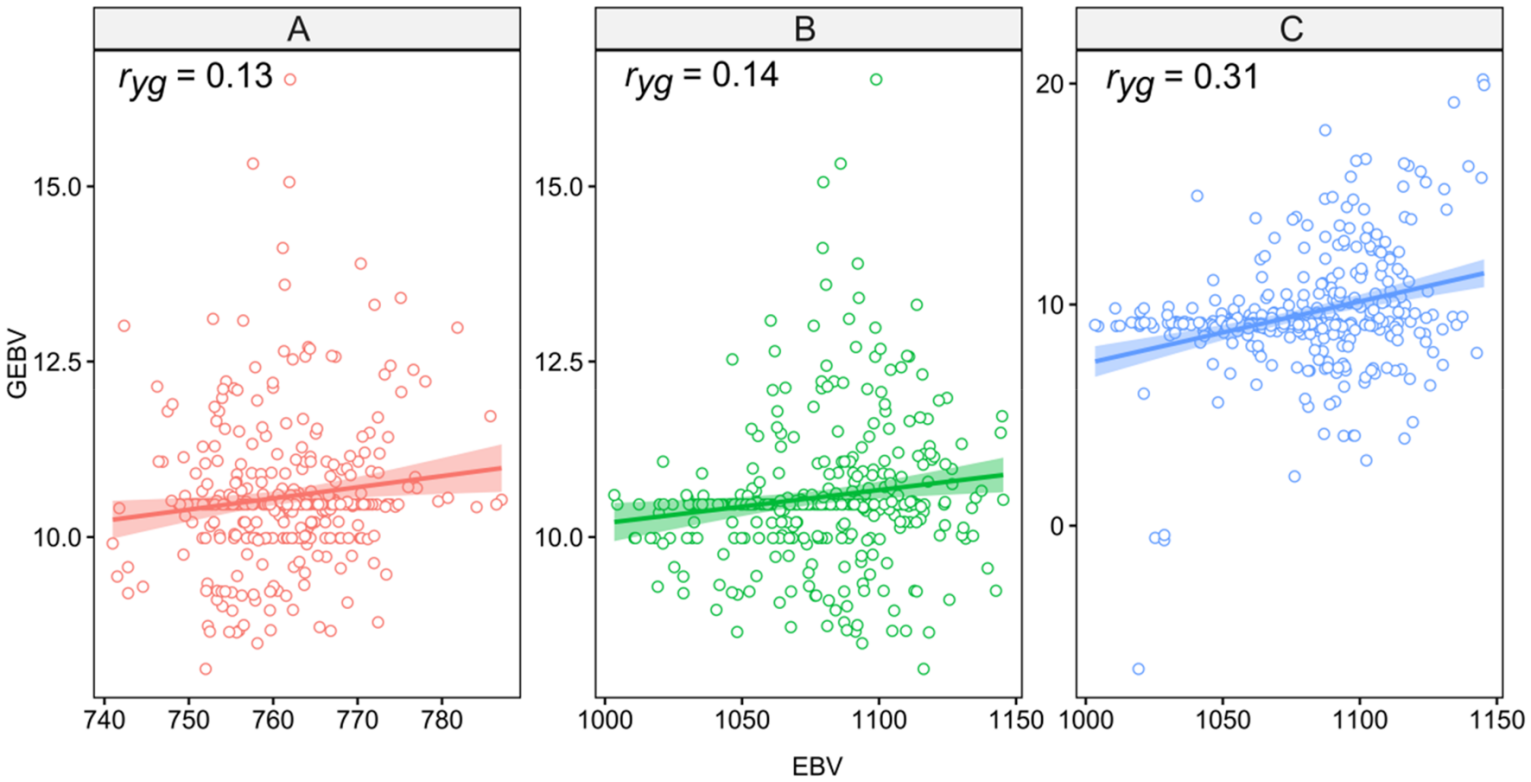
Accuracy of estimated prediction models in crop 1 validated in crop 2 (**A**), crop 1 validated in crop 3 (**B**), and crop 2 validated in crop 3 (**C**). The package used of R to create this Figure was ggplot2 (v0.3.3, https://cran.r-project.org/web/packages/ggplot2/index.html).

Predictive ability estimates of genomic selection (r_yg_) ranged from 0.27 to 0.57 for the first harvest and for average grain yield, respectively (Fig. 1). Jatropha demands at least 4 to 7 harvests for phenotypic selection with adequate accuracy. To verify if this time is also necessary for developing prediction models, we evaluated the accuracy of the models generated for fruit yield based on data collected in the first and second harvests, but validated in the same population, in the second and third harvests (Fig. 2).

Regarding the models developed for grain yield based on data collected in the first harvest and validated in the same population at 2 and 3 years old (second and third harvest, respectively), it can be observed that in the validation of the second harvest data the accuracy reduced more than 50% (Fig. 2A) in relation to the accuracy of the second harvest (Table 1). The same result can be observed when models estimated in the first harvest were validated based on data from the third harvest (Fig. 2B). However, moderate precision was verified when models estimated in the second harvest were used and validated based on data from the third harvest (Fig. 2C).

## Discussion

Based on narrow-sense heritability estimates (Table 1), the selection of superior Jatropha genotypes for grain yield based on phenotypic values will not provide selection gain for the next generation, since approximately 80% of the phenotypic variation is from a non-genetic origin. Grain yield, the main trait to be improved for biodiesel production, is very influenced by the environment. Other studies with Brazilian Jatropha genotypes have also found low heritability estimates over the harvests^18–21^. Thus, more accurate methodologies must be used to predict genetic effects. Therefore, GWS becomes more appropriate to select superior Jatropha genotypes than conventional methods based only on phenotypic data because this technique can efficiently capture small genetic differences between families.

However, even if the GWS allows adequate prediction of genetic effects, the heritability has importance in the model, because the lower the trait heritability the lower the phenotypic data accuracy and, therefore, lower the heritability of marker effects. Consequently, the lower will be the ability to reliably predict the phenotypes of individuals not sampled to compute the model. Several authors have demonstrated this theory through simulations^22–24^, in which increased trait heritability has resulted in increased GWS accuracy.

However, even with the impact of the low grain yield heritability throughout the harvests on selection, we can note that the predictive models performed well. This result corroborates those obtained by^22^, where accuracy increased only by 10–20% as heritability increased from 0.2 to 0.6, regardless of population size. Thus, unlike marker-assisted selection, GWS is efficient in selecting superior individuals even for low heritability traits as reported by^25^.

The observed values of accuracy based exclusively on phenotypic data showed low magnitude. This reveals that selection based only on phenotypic data has low accuracy in the first and second harvests, and moderate accuracy in the third harvests^26^. Similar findings were obtained by^27^, who found low accuracy for grain yield in Jatropha.

As for the GWS accuracy, low values were found in the first and second harvests, but the accuracy value was high in the third harvest. We highlight that these estimates were also obtained using genomic heritability as a proportion of phenotypic heritability, considering the efficiency of markers in capturing QTLs, i.e., considering the degree of imperfection of the linkage disequilibrium^26^.

The genomic selection efficiency depends on the correlation between the predicted and the actual genotypic value, i.e., the predictive capacity^26^. There was an increase in this correlation throughout the harvests, suggesting a higher additive genetic variation for this trait in this reproductive population. These findings show that r_gy_ values were higher for the harvests with the highest h^2^_a_ estimates. This indicates that phenotypes for low heritability traits contain higher environmental noise and hence will be less predictable by genomic models.

One of the principles of genomic selection is using a large number of markers able to cover the entire genome of the species to maximize the number of QTLs in linkage disequilibrium (LD) with at least one marker, allowing the maximization of the genetic variance explained by the QTLs^28^. However, the use of 811 SNPs allowed a satisfactory predictive ability. This result is consistent with those found by^29^ when assessing a Jatropha breeding population by GWS. These authors concluded that training models with 800 and 1000 markers are sufficient to capture the maximum genetic variation and hence the maximum predictive ability for grain yield in Jatropha families. In addition, not just the number of SNPs, but high LD with QTLs is the important factor in GWS success.

In genomic selection, the genotyping step is the costliest and often makes it impossible to apply genomic selection. However, given the results found here, as previously reported by^20^, reveal the possibility of using a small set of SNP markers in Jatropha breeding will enable significant gains in the short-term and at relatively low cost. The predictive ability of genomic selection (r_yg_) shows the potential of molecular information to consistently predict a phenotype^30^. The magnitudes obtained here were similar to those reported in forest species^24^ and coffee crop^31^.

Likewise^32^, by using canola as a model, reported that low-density marker sets comprising only a few hundred markers allow high accuracy of genomic prediction in breeding populations with strong linkage disequilibrium. The authors also mentioned that the breeder could obtain a significant advantage in selection by using reduced marker density, even when the prediction accuracy is lower than a high-density chip. This strategy provides substantial cost savings and thus enables phenotyping resources to be focused on pre-selected genotypes that, even with lower selection accuracy, will still allow significant gains for the breeding program.

Throughout the evaluation of the Jatropha harvests, many alleles will be acting with more or less expression on the expression of phenotypes. However, we do not know if the major effect alleles for grain yield in the first harvest will keep pronounced effect over the next harvests, i.e., over advanced ages. In this sense, considering that genomic selection requires phenotypic data for its calibration, a high correlation between the marker effects representing the alleles at different ages of the plant will allow the phenotype to be predicted earlier for prediction at future harvests. This would enable the early selection of superior genotypes by GWS.

Thus, grain yield was evaluated over three harvests, allowing the development of prediction models for each harvest. We tested how the models developed on the first harvest act in the prediction of phenotypes on the second and third harvests. However, the models developed for the first harvest showed limited accuracy in phenotype prediction in the second and third harvests. Given this result, we infer that there is a low genetic correlation between the first harvest and the second and third harvests. This low correlation is attributed to the lower yield stability of the Jatropha in the first harvest, since a significant part of the individuals in the population is still growing, making it impossible to express their productive potential.

When evaluating the agronomic performance of jatropha^33^, observed an increase in grain yield from 322 to 1972% from the first to the third harvest. On the other hand, the accuracy of the models developed based on the grain yield data of the second harvest was higher in the measurements of the third harvest, and there may be a higher genetic correlation between these harvests. Genotype by harvest interaction can affect the transferability of models between harvests when the first harvest is used. Therefore, the results obtained are essential to facilitate the ongoing use of genetic selection in Jatropha breeding programs.

Regarding the efficiency of wide-genome selection, considering the 50% reduction in the selection cycle, we observed an increased gain with GWS compared to the phenotypic selection of 66.74 and 346.45% for the second and third harvests, respectively. Our findings reveal that the use of GWS in Jatropha in an early manner is a reality to be explored. This strategy makes the applicability of GWS even more significant since late phenotyping would reduce the gain by time unit, i.e., the selection gain already achieved simply by conventional breeding.

By selecting superior individuals early through GWS analysis, the breeder will focus on potential genotypes and eliminating undesirable ones. For this reason, the costs of maintaining breeding populations in the field can be reduced. Furthermore, the early genomic selection makes it possible to carry out breeding populations with higher agronomic performance, which will maximize the genetic gains.

This possibility, as reported by^15^ and^34^, is due to the fact that genomic prediction and selection of superior genotypes can be performed at the seedling stage, and thus GWS becomes more time-efficient. Similar results were reported by several studies in pinus^35^, eucalyptus^15^, citrus^34^, and coffee^31^. An increase in breeding efficiency by using simulated data was also reported^36^.

Due to the significant population growth, the demand for energy sources, especially renewable energy, is intensified, since the use of energy from oil or coal is finite. Thus, encouraging the use of renewable energies, especially biofuels, has become one of the alternatives faced with the issue of global warming. Several studies have been carried out to consolidate new crops for biodiesel production.

In this sense, the use of Jatropha as a renewable energy source becomes a great alternative. However, to consolidate Jatropha as a new source of biodiesel, it is necessary to implement techniques able to assist the breeders in obtaining new cultivars, since the species is poorly improved. Genetic breeding programs have been started in several countries using conventional approaches to increase the grain and oil yield in Jatropha. However, by using conventional breeding, Jatropha yields are still low to make profitable and sustainable its growing^10,37^.

In this sense, clearing and using GWS allows access to genetic information, which is potentially useful to Jatropha breeding programs. Our study showed one of the first worldwide applications of GWS in a Jatropha breeding program. As Jatropha is a perennial crop, this tool becomes one of the most promising ways to promote the development of the crop for biodiesel production.

## Conclusions

The wide-genome selection proved to be promising for Jatropha breeding since it allows the accurate obtention of GEBV and higher efficiency in relation to phenotypic selection, making it possible to reduce the time needed for selection cycle.

In order to apply genomic selection in the first harvests, a large number of individuals in the breeding population are needed. In the case of few individuals in the population, it is recommended to carry out a higher number of harvests.

## Material and methods

### Breeding and genotyping population

A total of 386 individuals from the Jatropha breeding population, which comes from the crossing between 42 parents, were evaluated. The population was evaluated in random block design with six replicates, three plants per plot, and a spacing of 4 × 2 m. The parents come from the Active Germplasm Bank (BAG) of Embrapa Agroenergia, which was composed of genotypes from several regions. The grain yield (g plant^-1^) was evaluated in the years 2015, 2016, and 2017, corresponding to 1, 2, and 3 years old, respectively.

The DNA extraction was performed from fully expanded young leaves, according to the protocol of the manufacturer's manual *NucleoSpin Plant II* (Macherey–Nagel), with modifications. The quantification and quality of the samples were performed using the *NanoDrop Aspectrophotometer* to evaluate the A260/A280 wavelength ratio, which represents the amount of nucleic acids by the amount of protein in the sample. Samples with A260/A280 between 1.80 and 2.10 were considered adequate, indicating a low amount of protein and RNA in the samples. The samples were genotyped using the *Axiom myDesign Genotyping Arrays* platform, using a chip developed by Embrapa Agroenergia based on Affymetrix's Axiom technology (Axiom ENERCHIP) selected for species with bioenergetic potential. SNPs were filtered based on multiple criteria that included: (1) consensus sequence size, (2) minimum and maximum reading depth, (3) SNP quality score, (4) Minor allele frequency (MAF), (5) presence of other SNPs in the adjacencies, (6) SNPs present in various populations (if they were sampled), (7) SNPs present in coding regions, coverage of genes of interest (in this case genes related to biotic and abiotic stresses, acidification and oil biosynthesis) and (8) coverage at the genomic level (assessed by the distribution of SNPs in the reference genome).

Genomic data corresponding to 12,598 SNPs were submitted to an initial quality control (QC), where the marker exclusion criteria were: Call Rate = 0.95 and MAF = 0.04. The Call Rate is used to eliminate markers with a large amount of lost values, whereas MAF is related to the polymorphism of marker loci in the population. The critical level for the MAF parameter was obtained through the equation, $\text{MAF} = \frac{\text{1}}{\sqrt{\text{2N}}}$, where N refers to the number of genotypes in the population ^38^. After the QC, 811 SNPs were obtained that met the exclusion criteria.

### Predicting the genomic model and cross-validation

Genomic selection analyses were performed using the RR-BLUP random regression method^39^. All statistical modeling was performed using software R. RR-BLUP was performed using the rrBLUP package (mixed.solve function). For estimating the marker effects by RR-BLUP methodology, the following mixed linear model was used:

1 $${{y=X}}{\beta}+{{Za}}+{\epsilon}$$

wherein: ***y*** is the vector of phenotypic observations; ***β*** is the vector of fixed effects; ${a}$ is the vector of random marker effects; ***ε*** is the vector of random residuals; ***X*** and ***Z*** are the incidence matrices for ***β*** and *a*. The structure of means and variations in this model is described as defined by^40^:

$$ a~\sim~N\left( {0,~G} \right)\quad E\left( y \right) = X\beta  $$

$$ \varepsilon  \sim ~N\left( {0,~R = I\sigma _{\varepsilon }^{2} } \right)\quad Var\left( y \right) = V = ZGZ^{\prime } + R $$

$${G} = {I}{{\sigma}}_{{m}}^{{2}}$$

The genomic mixed model equations for predicting a by the RR-BLUP method is equivalent to:

2 $$ \left[ {\begin{array}{*{20}c}    {X{\prime }X}  \\    {Z{\prime }X}  \\   \end{array} ~\begin{array}{*{20}c}    {Z{\prime }X}  \\    {Z{\prime }Z + I\frac{{\sigma _{e}^{2} }}{{\sigma _{g}^{2} /n}}}  \\   \end{array} } \right]\left[ {\begin{array}{*{20}c}    {\hat{B}}  \\    {\hat{a}}  \\   \end{array} } \right] = \left[ {\begin{array}{*{20}c}    {X{\prime }y}  \\    {Z{\prime }y}  \\   \end{array} } \right] $$

wherein ${{\sigma}}_{{g}}^{{2}}$ is the total genetic variance of the trait.; ${{\sigma}}_{{e}}^{{2}}$ is the residual variance; *n*: number of markers. The prediction of the individual's genomic breeding value (GBV) is given by:

3 $${\widehat{{g}}}_{{j}} = {\sum }_{{i}}{{Z}}_{{ij}}{\widehat{{a}}}_{{i}}$$

The Z matrix was constructed from the number of alleles observed in each SNP marker (0, 1, or 2) and was standardized to have zero mean and variance 1, as described by^41^.

The k-fold cross-validation method was used. The set of observations was randomly divided into groups. In the analysis process, random samples of N_1_ = (9⁄10) × NT were used as the training population, while the remaining individuals in the population N_2_ = (1⁄10) × NT were used as the validation population, in which NT is the total number of individuals in the population. This process was repeated ten times (k = 10), using a different set of individuals as the validation population at a time. Thus, each fold did not overlap with the others, and at the end of the process (10 folds), all individuals had their phenotypes predicted by the genomic selection, as previously described by ^42,43^.

### Accuracy and efficiency of genomic selection

The accuracy of genomic selection was estimated according to^26^, in which it considers genomic heritability as a proportion of phenotypic heritability, thus considering the efficiency of markers in capturing QTL, i.e., it considers the degree of imperfection of linkage disequilibrium:

4 $$ r_{{g\hat{g}}}  = (r_{{yg}} )/\sqrt {\left( {h_{a}^{2} } \right)~x~(h_{g}^{2} )}  $$

wherein ${{r}}_{{yg}}$ is the predictive ability of GWS, obtained through the correlation between predicted breeding values and observed phenotypic values; ${{h}}_{{a}}^{{2}}$: is narrow-sense heritability, was obtained based on the following equation ${{h}}_{a}^{{2}}={\widehat{\sigma }}_{a}^{2} \slash {\widehat{\sigma }}_{p}^{2})$; and ${{h}}_{g}^{{2}}$ is the genomic heritability, was calculated as the ratio of the additive variance ${\widehat{\sigma }}_{a}^{2}$ to the phenotypic variance ${\widehat{\sigma }}_{p}^{2}$, $({{h}}_{g}^{{2}}={\widehat{\sigma }}_{a}^{2}/{\widehat{\sigma }}_{p}^{2})$.

The estimate of the number of individuals to be evaluated to achieve the desired accuracy was obtained by the expression:

5 $$ Ni~ = ~\frac{{r_{{g\hat{g}}}^{2} ~n_{{qtl}} }}{{(1 - r_{{g\hat{g}}}^{2} )h_{g}^{2} }}, $$

where in ${{r}}_{{g}\widehat{{g}}}$ it is GWS accuracy; n_qtl_ is the number of QTLs controlling each trait given by

6 $$  n_{qtl} = \frac{{(1 - r_{g\hat g}^2)Nh_g^2}}{{r_{g\hat g}^2}}, $$

N is number of individuals in the population and h^2^ genomic heritability^40^.

The selective efficiency of GWS compared to selection based on phenotypes only was calculated using the expression:

7 $${{IRPS}}_{{GWS}} = \frac{{{r}}_{{g}\widehat{{g}}}{{T}}_{{f}}}{{{r}}_{{y}\widehat{{y}}}{{T}}_{{GWS}}}{-1,}$$

wherein ${{r}}_{{g}\widehat{{g}}}$ is selective accuracy of GWS; ${{T}}_{{f}}$ is the average time for the selection cycle based exclusively on the phenotypes; ${{r}}_{{y}\widehat{{y}}}$ is the accuracy based on phenotypic selection, obtained through the equation: ${r}_{y\widehat{y}} = {(1-PEV/{\sigma }_{g}^{2})}^{1/2}$, where ${\sigma }_{g}^{2}$ is the genotypic variation of the population and PEV is the variance of the prediction error; ${{T}}_{{GWS}}$ is the average time for the selection cycle based on GWS^35^.

### Validating the models between harvests

The GWS models developed in each harvest were evaluated for accuracy in predicting breeding values, among other harvests. For this analysis, the accuracy was calculated by the correlation between GEBV derived from data collected in the first two harvests and EBV in the second and third harvests. As the same plant is compared between ages (harvests), there is a dependency between one plant on two different harvests. Therefore, tenfold cross-validation was performed as described previously. All analyses were performed in the R software ^44^.

### Statements

The authors are allowed to do research with Jatropha curcas in Brazil.

The handling of plants were carried out in accordance with relevant guidelines and regulations.
